# Assessment of treatment effectiveness, follow-up results, and recurrence patterns in children with *Helicobacter pylori* infection

**DOI:** 10.3389/fped.2026.1815432

**Published:** 2026-04-23

**Authors:** Mengde Luo, Danli Wei, Ling Jin, Yiling Wei

**Affiliations:** Department of Pediatrics, Liuzhou People’s Hospital, Liuzhou, China

**Keywords:** amoxicillin, clarithromycin, Helicobacter pylori, metronidazole, pediatric gastroenterology

## Abstract

**Background:**

*Helicobacter pylori* infection remains a prevalent chronic bacterial condition in children and is associated with significant long-term gastrointestinal complications, including peptic ulcer disease and an increased risk of gastric malignancy. Although standard triple therapy is widely used as first-line treatment, rising antimicrobial resistance and reinfection continue to challenge eradication success in children. This study aimed to evaluate the comparative effectiveness of two standard triple therapy regimens and to assess follow-up outcomes and recurrence patterns in children diagnosed with *H. pylori* infection.

**Materials and methods:**

This retrospective observational study was conducted at the Department of Pediatrics, Liuzhou People's Hospital, between January 2023 and December 2025. Children aged 4–13 years with confirmed *H. pylori* infection were included. Patients received either proton pump inhibitor (PPI) + amoxicillin + clarithromycin (*n* = 94) or PPI + metronidazole + clarithromycin (*n* = 34) for 10–14 days. Diagnosis and post-treatment assessment were performed using the ¹³C urea breath test (¹³C UBT), with histopathological confirmation in clinically indicated cases. Eradication was confirmed four weeks after completion of therapy. Follow-up evaluations were conducted at 6 and 12 months to monitor recurrence. Statistical analyses were performed using chi-square and independent t-tests, with *p* < 0.05 considered statistically significant.

**Results:**

A total of 128 children were included in the analysis. The overall eradication rate was 79.7%. The amoxicillin-based regimen demonstrated higher efficacy (84.0%) compared with the metronidazole-based regimen (67.6%). Recurrence occurred in 13.7% of successfully treated patients, predominantly among children younger than 10 years. Most recurrence cases were identified between 6 and 12 months following confirmed eradication.

**Conclusion:**

Amoxicillin-based triple therapy achieved superior eradication rates compared to metronidazole-based therapy in this children's cohort. However, recurrence remains a clinically relevant concern, particularly in younger children. Structured long-term follow-up and region-specific antimicrobial resistance surveillance are essential to optimize treatment outcomes in children's *H. pylori* management.

## 1 Introduction

*Helicobacter pylori (H. pylori)* infection is one of the most widespread chronic bacterial infections in children in the world, whose prevalence is markedly different among geographical zones, socio-economic layers, and age. Although it is mostly acquired in early childhood, its clinical manifestations tend to be subclinical, only appearing in later life as chronic gastritis, peptic ulcers, or, in some rare instances, as gastric malignancies (1). The prevention of *H. pylori* infection through early diagnosis and successful treatment of the infection amongst children is thus a very important issue of concern in public health, especially in high-endemicity and antibiotic-resistant areas (2).

The treatment strategy of *H. pylori* pediatric infection has significantly changed during the last twenty years. The rising cases of antimicrobial resistance, especially to clarithromycin and metronidazole, have questioned the effectiveness of the standard triple therapy, which consists of a proton pump inhibitor (PPI) and two antibiotics (3). Therefore, the diagnostics and treatment guidelines have now focused on customized therapy based on antibiotic susceptibility tests where possible. However, empirical therapy is still the default in most low-resource environments, and it commonly leads to less than optimal eradication rates (4).

According to the joint European Society for Pediatric Gastroenterology, Hepatology, and Nutrition (ESPGHAN)/ North American Society for Pediatric Gastroenterology, Hepatology, and Nutrition (NASPGHAN) guidelines for the management of *H. pylori* infection in children, diagnostic testing should generally be reserved for children with specific clinical indications rather than as part of a “test-and-treat” strategy used in adults. Testing is typically recommended in the presence of peptic ulcer disease or when endoscopic evaluation is clinically indicated. However, in regions with high infection prevalence and limited access to antibiotic susceptibility testing, empirical diagnostic and treatment approaches are still frequently applied in real-world pediatric clinical practice (5).

It is very important to follow-up on initial eradication treatment to confirm treatment success and check on potential reinfection or recrudescence. This is a special problem with children because their likelihood of reinfection is greater due to environmental exposure, behavioral determinants, and continuous risk among household contacts (6). Moreover, the absence of standardized follow-up intervals and the use of non-invasive non-diagnostic methods, including urea breath test (UBT) or stool antigen tests, provide some variability in the reported recurrence data. An assessment of the post-treatment results within a systematic follow-up design is crucial to enhance the rate of therapeutic success and create age-specific evidence-based management guidelines (7).

The study aimed to determine the effectiveness of treatment regimens against *H. pylori* in children in the real world and to follow up on the progress and recurrence patterns in the long run. This study will produce a rich clinical picture of a patient, incorporating demographic variables, patient response to treatment, diagnostic outcomes, and signs of recurrence, among others, to identify both short- and long-term effects of treatment (8). It is hoped that the findings will be used to improve children *H. pylori* treatment regimens, especially in high-endemic areas where resistance and reinfection have remained the enemy to treatment effectiveness (9).

## 2 Materials and methods

### 2.1 Study design and setting

The study presented is a retrospective observational study that will take place in the Department of Pediatrics, Liuzhou People's Hospital, from January 2023 to December 2025. The research was carried out to assess the effectiveness of treatment, follow-up outcomes after therapy, and recurrence rates of children with the diagnosis of *H. pylori* infection. Ethical consent was granted by the Institutional Review Board of Liu Zhou People's Hospital Medical Ethics Committee, No. KY2023-154-03. Conducting the study was done in strict compliance with the provisions of the Declaration of Helsinki. The necessity of informed consent was not met since the study was of a retrospective type, and the data about clinical cases were anonymized.

### 2.2 Study population and inclusion criteria

A total of 128 children aged between 4 and 13 years were included in the study based on a confirmed diagnosis of *H. pylori* infection.

Inclusion Criteria:

- Age ≤13 years
- Presence of upper gastrointestinal symptoms (e.g., abdominal pain, nausea, vomiting, or anorexia)
- Positive *H. pylori* diagnosis confirmed by either:
- ¹³C urea breath test (¹³C UBT), or
- Endoscopic biopsy with histopathological confirmation.

Exclusion Criteria:

- Prior *H. pylori* eradication therapy within the preceding 12 months
- Incomplete medical records
- Known immunodeficiency disorders
- Presence of concurrent chronic gastrointestinal diseases

### 2.3 Diagnostic tools

Initial diagnosis of *H. pylori* infection was established using the ¹³C UBT or upper gastrointestinal endoscopy with histopathological examination, depending on clinical indications.

The ¹³C UBT was performed in 115 children and served as the primary non-invasive diagnostic modality. The ¹³C UBT was performed following standard children's testing protocols. After an overnight fast, children ingested a ¹³C-labeled urea solution, and breath samples were collected before ingestion and 30 min afterward. The difference in ¹³CO₂ concentration between baseline and post-ingestion samples (Δ‰ value) was measured using infrared spectrometry. A cut-off value of Δ ≥ 4.0‰ was considered positive for *H. pylori* infection according to the manufacturer's recommended diagnostic criteria. Upper gastrointestinal endoscopy with gastric mucosal biopsy followed by histopathological confirmation was conducted in 13 clinically indicated cases, particularly in patients presenting with persistent or severe upper gastrointestinal symptoms. For post-treatment follow-up, eradication was confirmed four weeks after completion of therapy using the ¹³C UBT. Long-term surveillance for recurrence was conducted at 6 months and 12 months using the same diagnostic modality to ensure methodological consistency. Results falling close to the diagnostic threshold were carefully reviewed; however, no patients classified as successfully eradicated had borderline (“grey-zone”) values at the post-treatment assessment.

### 2.4 Treatment protocol

The first-line eradication therapy was based on standard triple therapy regimens, depending on the local antibiotic resistance as well as on individual clinical profiles. Most children (*n* = 94) received a combination of PPI (omeprazole 1 mg/kg/day in two divided doses), amoxicillin (50 mg/kg/day in two divided doses), and clarithromycin (15 mg/kg/day in two divided doses) during a period of between 10 and 14 days. The rest of the patients (*n* = 34) were put on a treatment regimen of PPI (omeprazole (1 mg/kg/day in two divided doses), metronidazole (20 mg/kg/day), and clarithromycin. The choice of regimens was based on the discretion of clinicians, previous history of antibiotic exposure, and the availability of the drugs. Medications were administered orally, and treatment adherence was assessed based on caregiver reports documented during follow-up visits and clinical record notes. Because this was a retrospective study, a standardized pill-count assessment was not systematically performed. None of the patients withdrew from the therapy, and general therapy was well tolerated. Follow-Up Protocol. Follow-up evaluations were scheduled at 4 weeks, 6 months, and 12 months after completion of therapy. Eradication was confirmed four weeks post-treatment using the ¹³C UBT. Recurrence was defined as a positive ¹³C UBT at 6 or 12 months following a previously documented negative result.

Success of eradication was defined as a negative ¹³C UBT performed 4 weeks after completion of therapy. Recurrence was established as a positive test in a patient who was previously eradicated in the follow-up of 6 months or 12 months.

### 2.5 Outcome measures

The primary outcome of this study was successful eradication of *H. pylori* infection, defined as a negative ¹³C UBT performed four weeks after completion of eradication therapy. Secondary outcomes included recurrence of infection at 6 months and 12 months among patients who had initially achieved successful eradication. Recurrence was defined as a new positive ¹³C UBT following a previously documented negative result. Additional secondary outcomes included the incidence of adverse drug reactions during treatment and the evaluation of demographic and clinical variables (including age, sex, and treatment regimen) potentially associated with eradication failure or recurrence. These outcome measures were selected to comprehensively assess both short-term therapeutic effectiveness and long-term infection dynamics in real-world children.

### 2.6 Statistical analysis

Statistical analysis was done with the SPSS software (IBM Corp., Armonk, NY). The demographic and clinical variables were statistically determined as descriptive statistics using mean (SD) as a continuous variable, and frequencies and percentages as a nominal variable. The chi-square test or Fisher Exact test was used to evaluate the relationships between the categorical variables, including the treatment regime and the eradication success or the recurrence rates. In the case of the continuous variables (age), the independent samples t-test or the non-parametric Mann–Whitney *U* test was used to compare the groups based on the distribution of the data. Subgroup analysis was conducted to evaluate recurrence rates according to age categories (<10 years and ≥10 years). A two-tailed *p*-value of <0.05 was considered statistically significant. Complete-case methodology was used to exclude all the missing data in the analysis.

## 3 Results

### 3.1 Patient demographics and clinical presentation

A total of 128 children diagnosed with *H. pylori* infection were included in this study. The age of the study population ranged from 4 to 13 years, with a mean age of 9.2 ± 3.1 years. There was a slight male predominance (53.9%). The most common presenting symptom was abdominal pain (78.1%), followed by nausea (45.3%), vomiting (31.2%), and loss of appetite (29.6%). No significant gender-based differences were observed in symptom distribution. The baseline demographic and clinical characteristics of the cohort are presented in Table 1. Patients were categorized into two treatment groups: 94 received PPI + amoxicillin + clarithromycin, and 34 received PPI + metronidazole + clarithromycin. All patients completed the initial treatment course without early discontinuation.

**Table 1:** Demographic and clinical characteristics of children with *H. pylori* infection (*n* = 128).

Parameter	Value
Total Patients	128
Age, years (mean ± SD; range)	9.2 ± 3.1 (4–13)
Male	53.9% (*n* = 69)
Female	46.1% (*n* = 59)
Abdominal Pain	78.1%
Nausea	45.3%
Vomiting	31.2%
Appetite Loss	29.6%
Treatment Group 1 (PPI + Amoxicillin + Clarithromycin)	94
Treatment Group 2 (PPI + Metronidazole + Clarithromycin)	34

*H. pylori*, *Helicobacter pylori*; SD, standard deviation.

### 3.2 Treatment outcomes and eradication rates

All 128 enrolled children completed their assigned *H. pylori* treatment regimens without early discontinuation. Of these, 94 patients received the standard triple therapy comprising a PPI, amoxicillin, and clarithromycin, whereas 34 were treated with a regimen consisting of a PPI, metronidazole, and clarithromycin. The choice of the antibiotic regimen was determined by physician preferences according to the history of the patient, previous exposure to the antibiotics, and the antimicrobial resistance patterns in the area.

**Figure 1:**
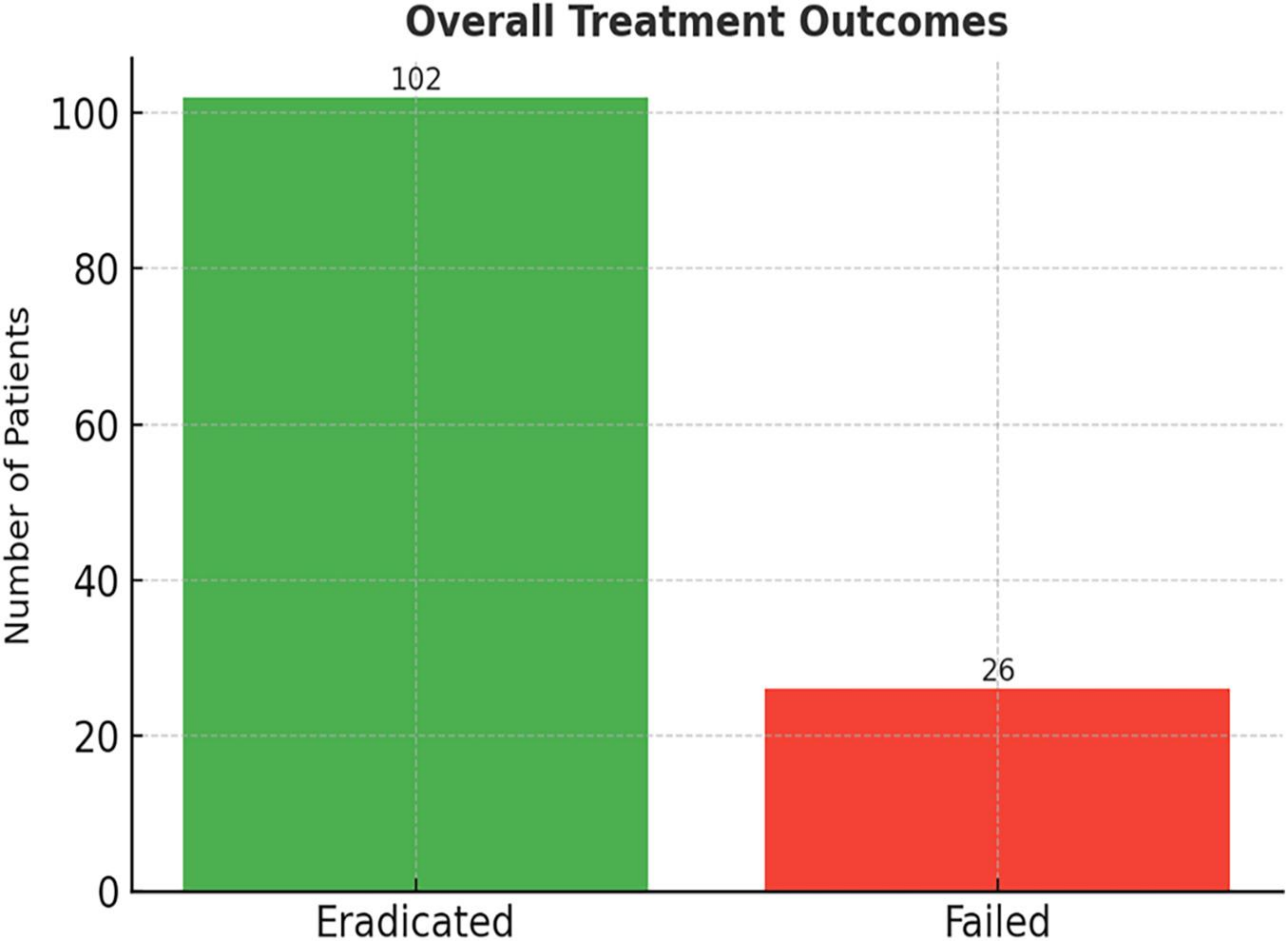
Flow diagram of children included in the study of *H. pylori* infection and treatment outcomes.

Eradication was assessed four weeks after completion of therapy using the ¹³C UBT in 102 children (79.7%). Out of the children that were treated using the amoxicillin-based regimen, 79 out of 94 children (84.0) obtained successful eradication. Conversely, 23 out of 34 children (67.6) in the metronidazole-based group were cured. The difference in eradication rates between the two regimens was statistically significant (*p* < 0.05). Table 2: Treatment Efficacy by Regimen: In comparison, Table 2 shows that the eradication rate and the lower rate of treatment failure in the amoxicillin-containing group were higher. The general distribution of the success and failure of treatment among the whole cohort is represented in Figure 1, and eradication is depicted as the most significant outcome.

**Table 2:** H. pylori eradication rates among children treated with amoxicillin- and metronidazole-based regimens.

Treatment Regimen	No. of Patients	Eradicated (*n*)	Eradication Rate (%)	Failures (*n*)
PPI + Amoxicillin + Clarithromycin	94	79	84	15
PPI + Metronidazole + Clarithromycin	34	23	67.6	11

*H. pylori, Helicobacter pylori*; PPI, proton pump inhibitor.

**Figure 2:**
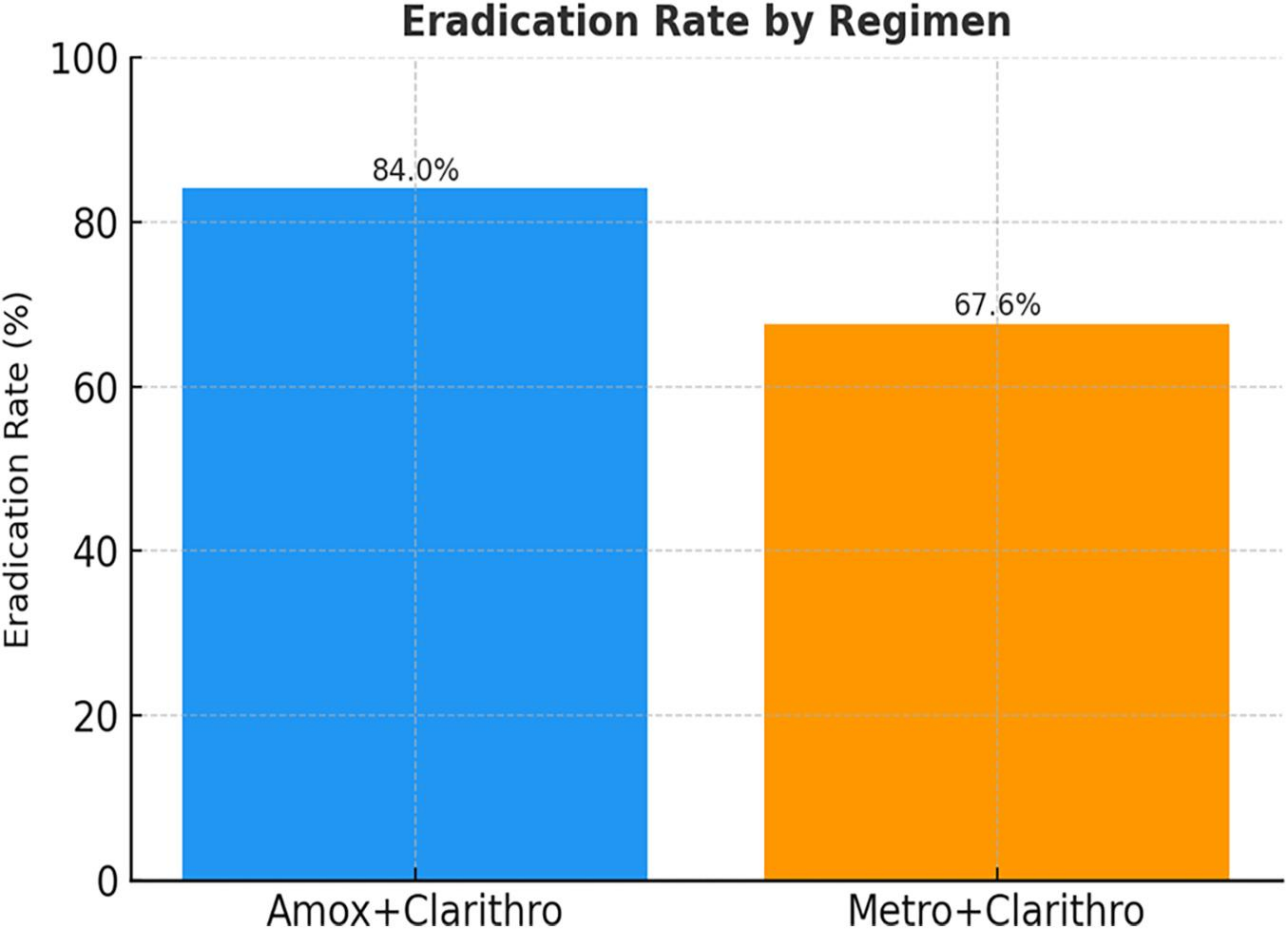
Comparison of *H. pylori* eradication rates between amoxicillin- and metronidazole-based treatment regimens in children.

Figure 1 shows the distribution of the eradication vs. failure rates in 128 children who received first-line therapy for *H. pylori*. Most patients (102) had been successfully eradicated, and 26 of them did not respond to initial treatment. In order to further explain the difference in treatment efficacy of the two regimens, Figure 2 is a visual representation of the eradication rates of the two regimens. The bar chart shows clearly that the PPI + Amoxicillin + Clarithromycin combination (84.0%) is the best in terms of performance when compared to the PPI + Metronidazole + Clarithromycin regimen (67.6%). The presented graphic illustration strengthens the statistical evidence and justifies the clinical guideline in favour of the amoxicillin-based treatment in the case of children, in which resistance is acceptable.

Comparison of treatment efficacy across two antibiotic regimens. Patients treated with amoxicillin-based triple therapy had a higher eradication rate (84.0%) than those treated with metronidazole-based therapy (67.6%), indicating a potential influence of antibiotic choice on treatment success.

### 3.3 Follow-up and recurrence

Among the 102 patients who reported a confirmed *H. pylori* eradication, 98 (96.1%) were followed up to 6 months, and 91 (89.2%) were followed up to 12 months. In this longitudinal observation, 14 patients were found to recur (13.7% of initially cured). Recurrence was confirmed by a ¹³C UBT, which was previously tested as negative after a negative result, following the recurrence of upper gastrointestinal symptoms. All patients classified as having successful eradication had clearly negative ¹³C UBT results at the four-week assessment, and no borderline or grey-zone results were observed among those who later developed recurrence. The pattern of recurrence also differed according to age. The children below the age of 10 years had a significantly higher rate of recurrence (17.3%) than their counterparts above 10 years (10.6%). The above findings are summarized in Table 3, which gives age-stratified recurrence data at both 6 and 12 months after eradication.

**Table 3:** Recurrence rates of *H. pylori* infection during follow-up among children after successful eradication.

Age Group	6-month Recurrence (*n*)	12-month Recurrence (*n*)	Total Recurrence (%)
<10 years	5	9	17.3
≥10 years	3	5	10.6

**Figure 3:**
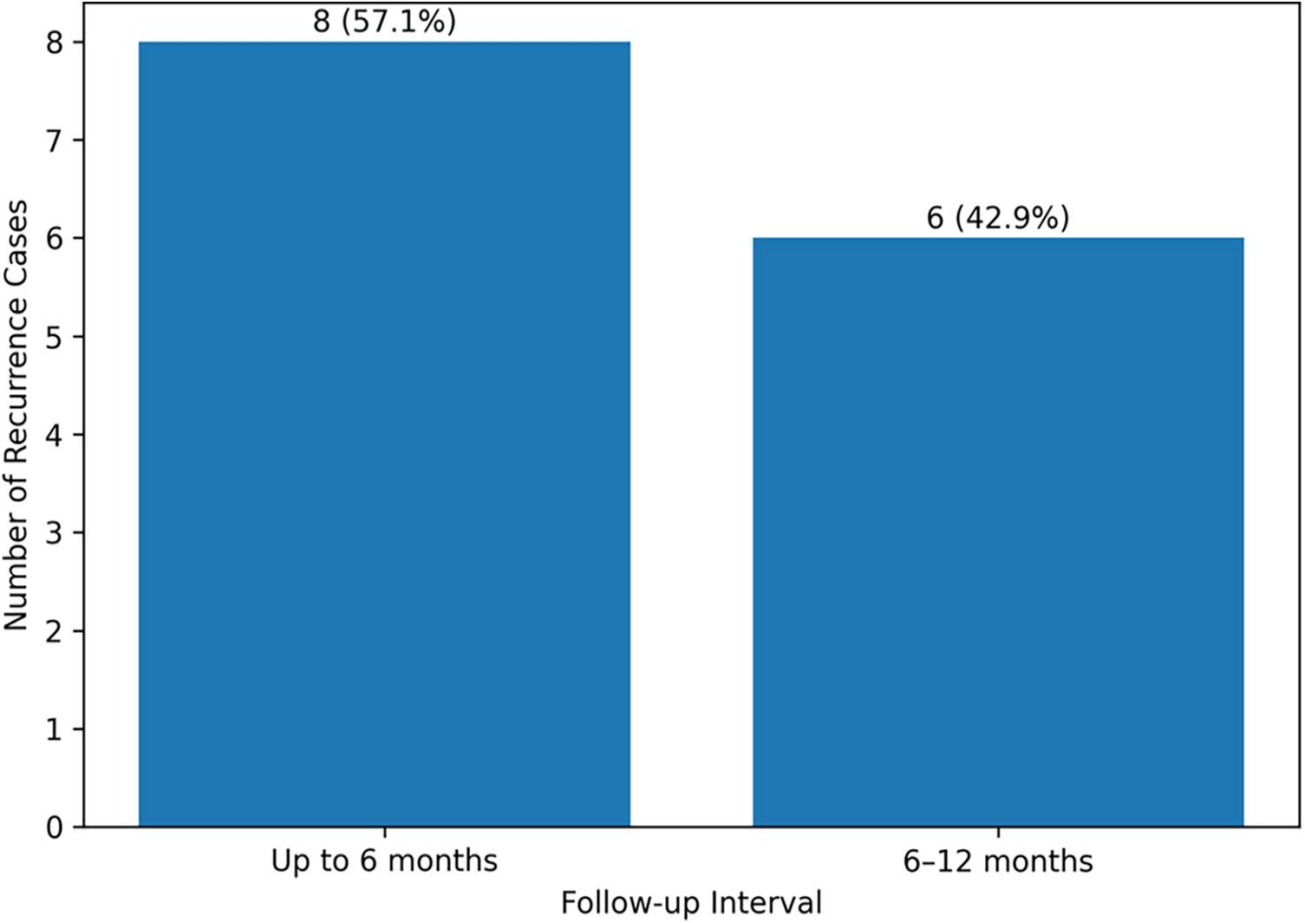
Recurrence pattern of *H. pylori* infection during follow-up in children after eradication therapy.

On a timeline, 8 cases (57.1%) of recurrence came after completion of treatment up to the 6-month evaluation, and 6 cases (42.9%) were found to have recurrence between 6 and 12 months. Within Figure 3, one can see this trend with a steady increase in recurrence during the 1-year follow-up period.

Sociodemographic information, including household income level and living conditions, was collected during follow-up. Although recurrence appeared more frequent among children from lower-income households and crowded living environments, these associations were not statistically significant in our analysis. Treatment regimen and probability of recurrence did not have a significant relationship (*p* > 0.05).

## 4 Discussion

This research shows that the overall eradication rate is high (79.7%) among children who are treated with conventional triple therapy to treat the infection of H. pylori. The regimen that included a PPI, amoxicillin, and clarithromycin demonstrated a higher eradication rate (84.0%) compared with the metronidazole-containing regimen (67.6%). However, this observation should be interpreted cautiously due to the relatively small number of patients in the metronidazole-treated group (Table 2; Figure 2). This disparity indicates the clinical importance of antibiotic choice, especially in children's groups where empirical regimens are still prevalent, because of the lack of access to resistance profiling.

These results align with global trends. Indicatively, when a large multicenter study performed by Adachi et al. (10) claimed higher eradication success with clarithromycin-based triple therapy, the use of amoxicillin was noted to be more successful, and failure rates were observed to be above 30% in metronidazole-based regimens, especially in areas with increasing resistance. These findings suggest a possible difference in eradication efficacy between the two regimens; however, the smaller number of children in the metronidazole-based group should be considered when interpreting these results. Likewise, a recent study by (11) had confirmed eradication rates of over 80% in children not previously exposed to any antibiotics when using PPI-Amoxicillin-Clarithromycin regimens. These relative results support the effectiveness of amoxicillin-based treatment as the first line of treatment as long as local clarithromycin resistance is not too high (more than 1,520%) (12). Our cohort eradication rate of 79.7% was lower than the ≥90% efficacy threshold recommended by the Maastricht VI consensus guidelines. This gap can be interpreted with the increase in antimicrobial resistance of microbes worldwide and supports the significance of the region-specific surveillance and individual regimens, especially among children (13).

Prolonged follow-up on children continued to assert that the recurrence of the *H. pylori* infection is a clinically significant issue (13). Recurrence in the present study happened in 14 among 102 children (13.7%), and most of them took place during the 6- and 12-month follow-up period (Figure 3). The rate of recurrence was significantly more common with children under 10 years of age (17.3%) compared to older children aged 10 or more years old (10.6%), as it is demonstrated in Table 3. This disparity between ages is probably the difference in the maturity of the immune system, hygiene practices, and environmental exposure. Such recurrence rates are generally in line with those that have been documented in the recent global studies (14) reported a 12-month recurrence rate of 12.5 among children who had been eradicated successfully, and that the younger age and household crowding were considered as the key risk factors. Similarly, a Japanese cohort study (15) of 180 children found a recurrence rate of 15.2% within one year, with most of the cases due to reinfection as opposed to treatment failure based on strain genotyping.

Interestingly, this is also in concord with the hypothesis that reinfection, particularly in high prevalence environment than the result of persistent bacteria recrudescence, is more likely to cause recidivism in children. The larger percentage of recurrence cases occurred after a period of more than 6 months, hence the necessity of the following initial eradication confirmation at four weeks post-therapy (16). Our repetition findings also agree with the findings of a 2023 review of World Journal of Current Medical Issues reporting the significance of 6 and 12 months post-discharge testing in children to capture the reinfection window (17). Although there was nothing statistically significant between treatment regimen and recurrence rate in our study (*p* > 0.05), socioeconomic factors, including overcrowding and low household income, were more prevalent among recurring cases. These results indicate that behavioral and environmental variables can be more predictive of relapse than the pharmacologic characteristics of the initial regimen, which supports the idea that *H. pylori* management in a pediatric environment is multidimensional.

The results of the given research have a great clinical implication on the population of pediatric gastroenterology, especially those working in resource-constrained environments, where empirical treatment of *H. pylori* is the norm. The significantly high success rate of eradication with the amoxicillin-containing regimen (84.0%), compared to that of metronidazole-based therapy (67.6%), particularly in the setting where clarithromycin resistance is moderate, indicates its superiority over the latter. These findings are consistent with recent ESPGHAN/NASPGHAN and Maastricht VI guidelines, which propose the use of amoxicillin in triple therapy in the first line in cases where evidence of resistance indicates the effectiveness (18, 19).

Moreover, the prevalence of 13.7% within a year, albeit within anticipated international limits, leads to concern about the risk of re-infection in children's age groups, particularly below the age of 10 years (Table 3; Figure 3). According to a systematic review conducted by (20), the recurrence rate of these diseases in children is 10%–20%, usually linked to socioeconomic determinants, including overpopulation in the family, insufficient hygiene, and inaccessibility of medical facilities. Our results are reflective of this trend and also point to the fact that treatment is not enough, but it is important to address the greater environmental factors of reinfection. Although the total eradication rate of our study (79.7%) is encouraging, it is the ≥90% efficacy threshold that is set by international standards of what can be considered an acceptable therapeutic outcome. To improve eradication success, alternative treatment strategies have been recommended in recent guidelines. These include bismuth-based quadruple therapy, susceptibility-guided therapy based on antibiotic resistance testing, or optimized combination regimens such as sequential or concomitant therapy. Implementing these strategies may help achieve eradication rates closer to the ≥90% target recommended by international consensus guidelines. Resistance testing and adjunctive approaches are also valuable and have been shown to eliminate the disease in East Asia and Southern Europe (21, 22), but are not yet fully implemented in our environment because of cost and access limitations.

Moreover, the prevalence of 13.7% within a year, albeit within anticipated international limits, leads to concern about the risk of re-infection in children's age groups, particularly below the age of 10 years (Table 3; Figure 3). According to a systematic review conducted by (23), the recurrence rate of these diseases in children is 10%–20%, usually linked to socioeconomic determinants, including overpopulation in the family, insufficient hygiene, and inaccessibility of medical facilities. Our results are reflective of this trend and also point to the fact that treatment is not enough, but it is important to address the greater environmental factors of reinfection. Although the total eradication rate of our study (79.7%) is encouraging, it is lower than the ≥90% efficacy threshold that is set by international standards of what can be considered an acceptable therapeutic outcome. Resistance testing and adjunctive approaches are also valuable and have been shown to eliminate the disease in East Asia and Southern Europe (24, 25), but are not yet fully implemented in our environment because of cost and access limitations. In addition, treatment adherence was evaluated based on caregiver-reported information documented in medical records, which may introduce reporting bias due to the retrospective nature of the study. Another limitation of this study was the unequal distribution of treatment regimens, with a smaller number of patients receiving the metronidazole-based therapy. This imbalance may limit the statistical strength of comparisons between the two regimens. Another limitation of the study is that improvement in gastrointestinal symptoms following eradication therapy was not systematically quantified. Although symptom relief was reported in many patients during follow-up visits, symptom resolution was not formally evaluated as a primary outcome measure.

## 5 Conclusion

This study evaluated the effectiveness of two commonly used triple therapy regimens for the treatment of *H. pylori* infection in children and assessed recurrence patterns during a 12-month follow-up period. The amoxicillin-based triple therapy demonstrated a higher eradication rate compared with the metronidazole-based regimen; however, the overall eradication rate remained below the ≥90% target recommended by international guidelines. Recurrence was observed in a proportion of successfully treated patients, particularly among children younger than 10 years, highlighting the importance of continued follow-up after eradication therapy. The findings also suggest that environmental and socioeconomic factors may contribute to reinfection risk in pediatric populations. These results should be interpreted cautiously due to the retrospective single-center design and the limited sample size in some treatment groups. In accordance with current pediatric guidelines, testing and treatment for *H. pylori* should be reserved for children with appropriate clinical indications rather than routine screening. Future multicenter prospective studies incorporating antibiotic resistance testing and optimized treatment strategies are needed to improve eradication outcomes in pediatric patients.

## Data Availability

The datasets used and/or analyzed during the current study were available from the corresponding author on reasonable request.
